# A supergene controlling social structure in Alpine ants also affects the dispersal ability and fecundity of each sex

**DOI:** 10.1098/rspb.2024.0494

**Published:** 2024-06-12

**Authors:** Ornela De Gasperin, Pierre Blacher, Solenn Sarton-Lohéac, Guglielmo Grasso, Mia Kotur Corliss, Sidonie Nicole, Sarah Chérasse, Serge Aron, Michel Chapuisat

**Affiliations:** ^1^ Department of Ecology and Evolution, University of Lausanne, Lausanne 1015, Switzerland; ^2^ Red de Ecoetología, Instituto de Ecología, A. C., Xalapa, Veracruz 91073, Mexico; ^3^ University of Manchester, Manchester M13 9PL, UK; ^4^ Universite libre de Bruxelles, Brussels 1050, Belgium

**Keywords:** sociality, dispersal, supergenes, dominance, polymorphism, *Formica selysi*

## Abstract

Social organization, dispersal and fecundity coevolve, but whether they are genetically linked remains little known. Supergenes are prime candidates for coupling adaptive traits and mediating sex-specific trade-offs. Here, we test whether a supergene that controls social structure in *Formica selysi* also influences dispersal-related traits and fecundity within each sex. In this ant species, single-queen colonies contain only the ancestral supergene haplotype *M* and produce *MM* queens and *M* males, while multi-queen colonies contain the derived haplotype *P* and produce *MP* queens, *PP* queens and *P* males. By combining multiple experiments, we show that the *M* haplotype induces phenotypes with higher dispersal potential and higher fecundity in both sexes. Specifically, *MM* queens, *MP* queens and *M* males are more aerodynamic and more fecund than *PP* queens and *P* males, respectively. Differences between *MP* and *PP* queens from the same colonies reveal a direct genetic effect of the supergene on dispersal-related traits and fecundity. The derived haplotype *P*, associated with multi-queen colonies, produces queens and males with reduced dispersal abilities and lower fecundity. More broadly, similarities between the *Formica* and *Solenopsis* systems reveal that supergenes play a major role in linking behavioural, morphological and physiological traits associated with intraspecific social polymorphisms.

## 1. Introduction

Dispersal shapes patterns of genetic relatedness within and between social groups [1,2]. Dispersal therefore coevolves with multiple social traits (reviewed in [3]), including social group living [4], number of breeders per group [5], division of labour [6] and production of public goods [7]. Dispersal also typically covaries with fecundity [8] and sex allocation [9]. If alternative dispersal traits covary with multiple other characters within a species, an immediate question that arises is what prevents maladaptive combinations of traits from occurring?

One possible solution for generating intraspecific polymorphisms that adaptively combine multiple traits is to link co-adapted alleles in supergenes [10]. Supergenes are non-recombining genomic regions that collectively produce discrete multi-trait phenotypes, like sexes, ecotypes and social forms [11–13]. A theoretical model predicted that social polymorphism arises from the coevolution of dispersal and social behaviour, through genetic linkage between alleles for the two types of traits [14]. Supergenes are, therefore, prime candidates for co-determining social, dispersal and other life-history traits, and for preventing the emergence of maladaptive combinations of traits. However, traits can also be correlated in indirect ways, without genetic linkage. For example, the social environment may indirectly influence the development and behaviour of an organism. Hence, correlations between traits may be due to both genetic and environmental covariation.

Ants are ideal systems to study if and how supergenes co-determine forms of social organization, dispersal and other life-history traits, because supergenes control colony social structure in at least five independent ant lineages (*Solenopsis*, *Formica*, *Leptothorax*, *Cataglyphis*, *Pogonomyrmex*) [15,16]. Moreover, in ants, colony social structure is generally associated with alternative dispersal and reproductive strategies [5,17,18]. Specifically, colonies with a single reproducing queen (monogyne colonies) tend to exhibit higher dispersal, independent colony establishment by queens and higher per capita fecundity, while colonies with multiple reproducing queens (polygyne colonies) typically display limited dispersal, queen philopatry, colony budding and reduced per capita fecundity. Alternative dispersal strategies (e.g. fly away individually and establish independent colonies, or stay in the natal nest and establish novel nests by dispersing on foot with workers) are typically associated with morphological, physiological and behavioural differences [17]. Hence, supergenes in ants may directly determine alternative forms of social organization (e.g. number of queens per colony), dispersal, fecundity and other life-history traits.

The two best studied social supergenes in ants, in the fire ant *Solenopsis invicta* and in the Alpine silver ant *Formica selysi*, share many similarities [15,16]. This suggests that the two independently evolved supergenes have captured alleles underlying similar traits in the two clades [19]. In each lineage, a derived supergene haplotype (called *Sb* in *S. invicta* and *P* in *F. selysi*) is necessary and sufficient for making the colony polygyne [16]. In both cases, the derived haplotype is dominant for polygyny, in the sense that females (queens and workers) carrying at least one copy of this haplotype establish polygyne colonies, while females lacking it establish monogyne colonies [19–22]. Moreover, in both lineages, the derived haplotype differs from the ancestral haplotype by several large inversions (which halts recombination between the two haplotypes) [23,24], has recessive deleterious effects [25,26] and displays selfish properties, albeit through different mechanisms [27,28]. Moreover, the two social supergenes are associated with alternative dispersal tactics and other correlated life-history traits.

In both *S. invicta* and *F. selysi*, the monogyne social form has higher dispersal tendencies than the polygyne social form. Each year, monogyne colonies produce more winged queens and males than polygyne colonies [29–32]. The monogyne queens are also larger and more fecund than polygyne queens [20,29,30,33]. Furthermore, in *S. invicta*, the heavier *BB* queens are more attracted to open patches than *Bb* and *bb* queens [33], and *B* males produce more sperm cells than *b* males (males are haploid) [34]. In both species, mating between social forms is non-random, which affects gene flow between social forms and contributes to the maintenance of polymorphism [15,22,29,35,36]. Finally, in *F. selysi*, relatedness patterns indicate that polygyne queens are more philopatric than monogyne queens, mating with more related males, being occasionally reaccepted in their maternal nest and possibly dispersing on foot with workers [22]. These findings support the theoretical prediction that social polymorphisms can arise when alleles underlying social, dispersal and other life-history traits are linked in supergenes [12,14], and indicate that supergenes have similar roles across independent clades.

**Figure 1.**
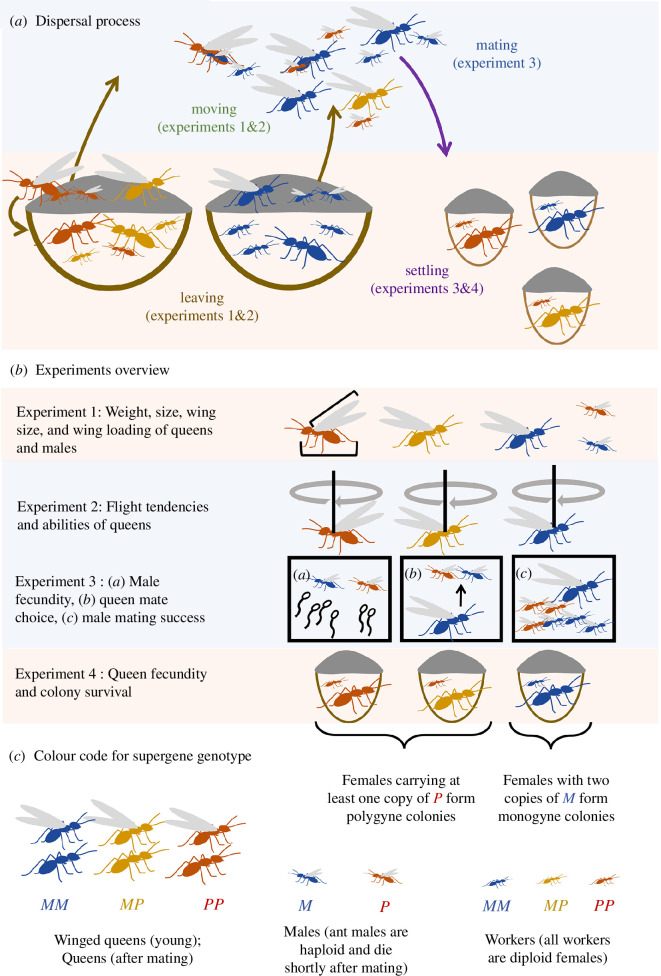
Overview of the dispersal process of genetically determined social forms of *F. selysi* (*a*) and experiments carried out for each step of the dispersal process (*b*), with colours indicating the social supergene genotype of queens, males and workers, respectively (*c*). Effective gene flow between social forms, and the spread of each social form, depend on the number of colonies producing winged queens and males, and their sex allocation [29,32,37], the dispersal strategies of queens and males ([22] and experiments 1 and 2), the distance travelled before and after mating (experiments 1 and 2), the mating tactics of queens and males (experiment 3), the fecundity of queens and survival of their colonies ([26,38,39] and experiments 3 and 4) and the colony phenotype of each cross [19,22]. Different colours match each supergene genotype (blue: *MM/M;* orange: *MP;* red: *PP/P*).

**Figure 2.**
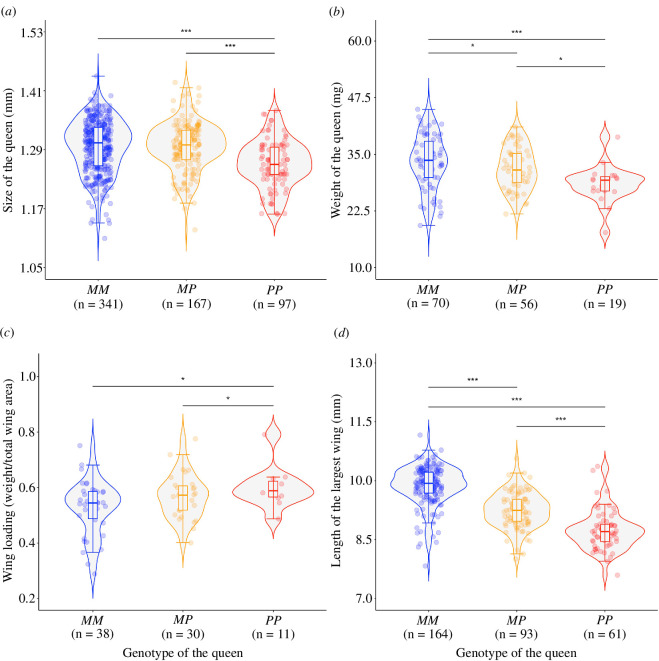
Queens with alternative social supergene genotypes differed in morphological traits. Size (distance between the eyes) (*a*), weight (*b*), wing loading (*c*) and length of the largest wing (*d*) of queens of alternative genotypes. Each point represents a queen. The horizontal line inside each box shows the median value per queen genotype, and the data inside the boxes includes the middle 50% of the data. Significance levels: *p < 0.05, ***p < 0.001 after false-discovery rate adjustment for multiple comparisons.

Effective gene flow within and between social forms, and the spread of each social form, depend on dispersal, mating, fecundity and fitness of individuals with alternative supergene genotypes (figure 1). Supergenes could affect multiple morphological, physiological and behavioural traits that in turn influence the success of individuals at each step of the dispersal process (leaving the natal ground, moving and settling in a new site; figure 1; [2]). Supergenes could also mediate sex-specific trade-offs. For example, carrying large amounts of nutrients may help a queen establish an independent colony and raise her first cohort of workers [40] but may also lower her ability to fly long distances, provided she has a similar wing area, due to the costs and biomechanical constraints of carrying these extra nutrients [41]. This trade-off will not apply to male ants, as they do not found colonies. Although we know that supergenes covary with forms of social organization, dispersal and fecundity in ants, we still lack a holistic view of how social supergenes affect each step of the dispersal process (leaving–moving–mating–settling; figure 1). In many cases, we also do not know if supergenes have direct or indirect genetic effects on dispersal, fecundity or other life-history traits, nor do we know if they influence sex-specific trade-offs.

To answer these questions, we investigated whether and how the social supergene affects the morphology, behaviour and fecundity of each sex of the socially polymorphic Alpine silver ant, *F. selysi*. We evaluated the effect of the supergene on traits of males and queens that influence each step of the dispersal process, from leaving the natal nest to settling in a new site (figure 1). We carried out a series of integrated experiments involving over 3000 queens and males. *F. selysi* is useful for investigating the impact of supergenes on dispersal and fecundity, for three reasons. First, a well-characterized supergene controls colony social structure [19,22,23]. Second, genome-wide genetic analyses have shown stronger genetic differentiation between populations for the polygyne than for the monogyne social form, suggesting higher dispersal by the monogyne form [42]. Yet, the mechanisms accounting for differences between social forms in effective gene flow remain largely unknown [29]. Finally, as heterozygote and homozygote queens are emitted by the same polygyne colonies, we can disentangle direct genetic effects of the supergene from indirect genetic effects due to other colony members [43,44]. Altogether, we found that the *F. selysi* social supergene affects dispersal and fecundity of both sexes, and we uncovered direct genetic effects of the supergene. Hence, independently evolved supergenes in fire ants and Alpine silver ants similarly affect multiple social, dispersal and life-history traits.

## 2. Methods

### (a) General methods: study species, field collection, ant rearing and genotyping

The Alpine silver ant, *F. selysi*, is a socially polymorphic species occupying riverine habitats in Alpine valleys [37,45]. Colony social structure is controlled by a large (13.8 Mb long, ~745 genes) and ancient (~30-million-year-old) supergene [19,23]. The single reproductive queen in monogyne colonies has the *MM* supergene genotype and is mated to one or two *M* male(s), so that all individuals produced by these colonies only carry the *M* haplotype. In contrast, females in polygyne colonies have one or two copies of the derived *P* haplotype [19,22]. The polygyne colonies produce workers, new queens and males carrying at least one copy of the *P* haplotype. Indeed, *MM* daughters and *M* sons fail to develop in these colonies, because the *P* haplotype in queens selfishly distorts the transmission ratio through maternal effect killing [27]. On average, monogyne colonies have 3000 workers and a lifespan of 10 years, while polygyne colonies have 30 000 workers and a lifespan of 30 years [32]. Queens and males of all genotypes fly to mating aggregations, but most queens are emitted by the monogyne social form [29]. *MM* queens mate mostly assortatively with respect to social form [22,29,38,46], despite the high abundance of *P* males [29]. The proximate mechanisms accounting for this pattern of non-random mating are not yet known [38].

We collected winged queens, males and workers of *F. selysi* as described in [39]. We sampled ants from two populations in Valais, Switzerland, throughout four summers (2017–2020; Derborence: 46.2806° N, 7.2157° E, 1450 m above sea level (a.s.l); Finges: 46.3138° N, 7.6012° E, 400 m a.s.l.). Colony fragments were inspected twice a week to detect and separate emerging queens and males. We kept colony fragments with water and food ad libitum, at 25 ± 3°C, with 70% humidity in a 12:12 h light:dark cycle. In total, we sampled queens from 167 (97 monogyne and 70 polygyne) unique colonies and males from 119 (80 monogyne and 39 polygyne) unique colonies (some of these unique colonies were sampled across multiple years). We genotyped the social supergene of three workers per colony, to determine colony social form, following [29]. Briefly, we applied a quantitative polimerase chain reacion (qPCR) assay using TaqMan probes targeting three haplotype-specific single nucleotide polymorphisms (SNPs) of the supergene [29]. We also genotyped the supergene of all queens from polygyne colonies, using DNA extracted from their wings or legs. Wings were measured based on pictures taken with a Leica microscope, using a custom-made MATLAB script written by Santiago Herce Castañón (electronic supplementary material, image 1). All measurements and experiments were carried out blind to the social origin and genotype of all individuals. In each experiment, we used a minimum of 40 unique colonies for queens (maximum 136) and 33 unique colonies for males (maximum 69).

### (b) General statistical procedures

All analyses were conducted using R version 4.1.1 [47], with ‘glmer’, ‘lmer’ and ‘glm’ functions [48], unless otherwise stated. Model estimates, standard errors (SE) and *p* values were obtained using the ‘summary’ or ‘anova’ functions [47]. We used type II sum of squares for models without interactions and type III for models with interactions [49]. We evaluated model residuals to ensure they had normal distributions, displayed no patterns, had similar variance between factors, through time and across values of continuous co-variables, following [49]. *Post hoc* comparisons were obtained with the ‘lsmeans’ function [50] with the false discovery rate (FDR) *p* correction method. We controlled for the population of origin (Finges/Derborence) if we pooled data from individuals emerging from different populations, and for the queen (*MM*/*MP*/*PP*) and male (*M/P*) genotype. We controlled for non-independence of individuals by including their colony of origin as a random effect, nested within the experimental year (if we included individuals emerging from the same colonies but collected in different years).

### (c) Specific methods and analyses

#### (i) Experiment 1: supergene’s effect on queen and male morphology

We measured the fresh weight of 145 virgin queens (70 *MM*, 56 *MP*, 19 *PP*), estimated body size (distance between the eyes) of 605 queens (341 *MM*, 167 *MP*, 97 *PP*) and of 127 males (53 *M*, 74 *P*), wing size (length, width, perimeter and area; electronic supplementary material, figure S1) of 318 queens (164 *MM*, 93 *MP*, 61 *PP*) and of 407 males (236 *M*, 171 *P*), and estimated wing loading (fresh weight divided by the sum of the area of her four wings [51,52]) of 79 virgin queens (38 *MM*, 30 *MP*, 11 *PP*). We compared these measurements using generalized linear mixed models (GLMMs) with normal error distribution, except for queen’s weight and for male’s wing length, for which we used the ‘lme’ function to control for differences in the variance of the residuals between populations, using the *varIdent* variance structure [53].

#### (ii) Experiment 2: supergene’s effect on queens’ flight tendencies and abilities

We evaluated the flight propensity, flight abilities and costs of flying of 178 virgin queens (90 *MM*, 64 *MP*, 24 *PP*), using a custom-made mill (electronic supplementary material, figure S2) that automatically records flying time, speed and distance [54]. Queens tested were sampled randomly from their laboratory colony fragment and weighed on a microbalance (Mettler Toledo MT5) prior to and straight after testing. After each trial, queens were dry-frozen for supergene genotyping and body size measurements. Additional methods are described in the electronic supplementary material. We compared queens’ flight propensity with a GLMM with binomial error distribution (1 = the queen flew), using in one model the queen’s genotype and in a second model the queen’s weight (because queen weight and genotype were highly correlated; see results of experiment 1). We compared the weight lost by queens during the trial, using two GLMMs with normal error distribution. First, we considered all queens and included as explanatory variables whether she flew or not. Second, we included only queens that flew (and removed *PP* queens, as very few of them flew, but results were qualitatively similar if we included them) and included as an explanatory variable the time the queen spent flying (log-transformed). In both models, we included the trial duration (log-transformed) as a covariable. As only 13 *MM*, 9 *MP* and 3 *PP* queens flew, we did not compare speed nor distance.

#### (iii) Experiment 3: supergene’s effect on male fecundity and mating success

*Male fecundity*. We estimated the number of sperm cells produced by 57 males (20 *M*, 37 *P*) and the number of sperm cells that 36 males transfer to queens during their first (11 *M*, 11 *P*) and second (7 *M*, 7 *P*) mating event, respectively, using flow cytometry [55,56], as described in the electronic supplementary material. We genotyped all males and estimated the size (distance between the eyes) of virgin males. We compared the absolute number of sperm cells produced by *M* males and *P* males (collected from the males’ accessory testes), including his size (the distance between his eyes) as an explanatory variable. We compared the number of sperm cells collected from the queen’s spermatheca, using as a response variable the absolute number of sperm cells and as explanatory variables the male’s mating condition (first/second mating), and the interaction between this and his genotype.

*Queen mate choice.* We compared *MM* queen’s preference for the odour of *M* or *P* males (experiment 3b described in the electronic supplementary material), by placing virgin queens to interact with the odour extracted from males (most *MM* queens mate with *M* males despite the high abundance of *P* males and no clear preference towards *M* males in mating trials). To assess whether queens could recognize the odour of a male, we compared the time (in seconds) that 39 *MM* queens spent near the odour of a male or of the solvent (control). Then, we compared the time (in seconds) that 54 *MM* queens spent near the odour of *M* or *P* males. We fitted models with Poisson error distribution, included an observation-level random effect to account for overdispersion [57], and, in the second model, added whether the odour came from a virgin or mated male and the interaction between these factors as explanatory variables. In both models, we added the queen’s ID as a random effect and removed her colony of origin as it explained zero variance.

*Male mating success.* We evaluated the mating success of 872 virgin males (393 *M*, 479 *P*) in 37 experimental mating boxes, with a ratio close to 1:1 of *M*:*P* males, competing for a limited number of *MM* queens, in artificial swarms (*MM* queens account for 90% of queens in natural swarms; see the electronic supplementary material). We also evaluated the mating success of 149 previously mated males (70 *M*, 79 *P*). In these trials, we placed two *MM* virgin queens and four previously mated males (2 *M* and 2 *P*), in 39 experimental boxes (multiple mating by *M* males could explain the assortative mating observed in nature of *MM* queens to *M* males [22,29], despite no evident temporal or spatial segregation of males in the wild [29], similar numbers of *M* and *P* males in the wild [29] and no clear queen mate choice [22]). We compared the mating success of males using a GLMM with binomial error distribution (1 = the male mated). We included as fixed factors his mating condition and the interaction between this and his genotype. We included the proportion of queens to males as a covariable and the experimental box as a random effect.

#### (iv) Experiment 4: supergene’s effect on queen fecundity and colony survival

We compared the survival and number of workers of 630 1-year-old colonies established by queens of each supergene genotype and mated to *M* or *P* males (colonies formed by a single queen mated to a single male). Detailed methods of queen mating and rearing are described in [39]. Briefly, we placed young queens to mate alongside non-nestmate virgin males during summer 2017. We placed each mated queen in a box with sand, food and water ad libitum. Queens went through an experiment that mimicked harsh or mild winters. After a year, we counted how many queens remained alive and the number of workers they had, and genotyped all polygyne queens. We compared the probability that queens would be alive after a year with at least one worker with a GLMM with binomial error distribution (475 *MM*, 88 *MP* and 67 *PP* queens). Then, for the 213 surviving queens with at least one worker (170 *MM*, 30 *MP* and 13 PP queens), we compared the number of workers present in each 1-year-old colony with a GLMM with Poisson error distribution. In the first model, we included the queen and male genotype as an explanatory variable, and the interaction between these variables. In the second one, we did not include the interaction due to the small sample size of *PP* queens, and we included an observation-level random effect (OLRE) to account for overdispersion.

## 3. Results

### (a) Experiment 1: supergene’s effect on queen and male morphology

**Figure 3.**
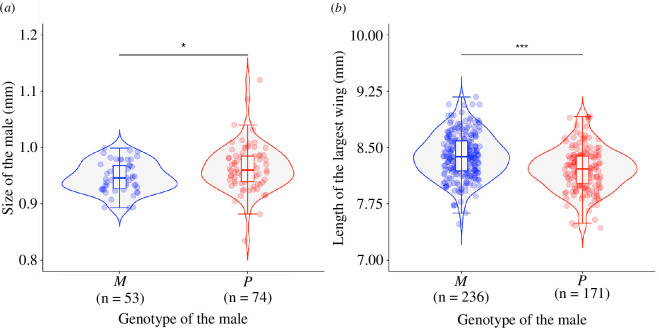
Males with alternative social supergene haplotypes differed in morphological traits. Size (distance between the eyes) (*a*) and length of the largest wings (*b*) of *M* and *P* males. Each point represents a male. The horizontal line inside each box shows the median value per male genotype, and the data inside the boxes includes the middle 50% of the data. Significance levels: *p < 0.05, ***p < 0.001 after false discovery rate adjustment for multiple comparisons.

Queens and males with alternative social supergene genotypes differed in all morphological traits studied (electronic supplementary material, table S2; figures 2 and 3). The effect sizes were larger for queens than for males, and they followed the same direction in both sexes except for body size. Furthermore, *PP* queens and *MP* queens differed in all morphological traits, which reveals that the supergene has a direct genetic effect on dispersal-related traits of queens. *PP* queens were the smallest (electronic supplementary material, table S1, model 1) and lightest (electronic supplementary material, table S1, model 2) queens. They also had the least aerodynamic bodies (with higher wing loading than *MM* and *MP* queens, electronic supplementary material, table S1, model 3) and had the smallest wings (electronic supplementary material, table S1, models 4 and 5). *PP* queens were 15% lighter than *MM* queens, and their total wing area was 23% smaller than that of *MM* queens. *MM* and *MP* queens had a similar size and wing loading (electronic supplementary material, table S1, models 1 and 3). However, *MM* queens were heavier (electronic supplementary material, table S1, model 2) and had larger wings (electronic supplementary material, table S1, models 4 and 5) than *MP* queens. Queen’s size and weight were highly correlated (Spearman’s *ρ* = 0.37, *p* < 0.0001).

*P* males were slightly larger than *M* males (electronic supplementary material, table S1, model 6, figure 3). In contrast, *M* males had bigger wings than *P* males (electronic supplementary material, table S1, models 7 and 8, figure 3). Size differences between *M* and *P* males were small. *P* males were only 1.1% larger than *M* males (the median size of *M* males was 0.946 mm and of *P* males was 0.96 mm), while the total area of the wings of *P* males was 6% smaller than those of *M* males, and the length of the largest wing of *P* males was 2% smaller than that of *M* males.

### (b) Experiment 2: supergene’s effect on queen flight tendencies and abilities

**Figure 4.**
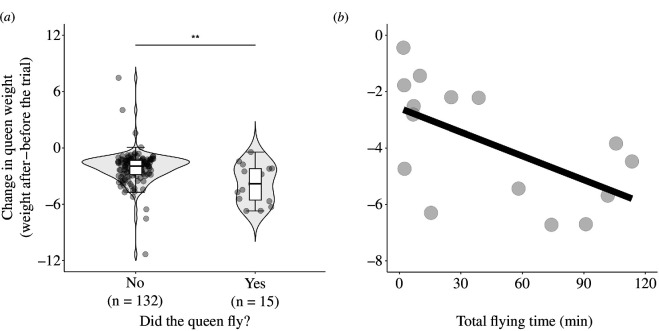
Flying is costly for queens. Queens that flew lost significantly more weight (in mg) than queens that did not fly (*a*). Slightly positive values indicate the queen gained weight during the trial, probably due to glue left on her thorax. The horizontal line inside each box shows the median value, and the data inside each box includes the middle 50% of the data. Significance levels: **p < 0.01. Queens that flew for longer periods of time lost significantly more weight (*b*). The graph shows a linear relationship between weight loss and time flying. (*b*) Only includes queens that did fly. In both plots, each point represents one queen.

Queens of alternative genotypes were equally likely to fly. Approximately 14% of *MM,* 14% of *MP* and 13% of *PP* queens flew (electronic supplementary material, table S2, model 9). Heavier queens were more likely to fly (model 10). Flying was costly for queens, as queens that flew lost on average twice as much weight as queens that did not fly (3.84 and 1.93 mg, respectively; electronic supplementary material, table S2, model 11, figure 4*a*). For queens that flew, the longer they flew for, the more weight they lost (electronic supplementary material, table S2, model 12, figure 4*b*). *MM* queens flew a median time of 15.45 min (range = 1.93–218.82) and covered a median distance of 259 m (range = 21.8–1957), and *MP* queens flew a median time of 25.26 min (range = 2.46–114.4) and a median distance of 409 m (range = 22.5–2291). The three *PP* queens flew for 2.28, 6.06 and 58 min and covered 23.80, 126.25 and 1084.04 m, respectively.

### (c) Experiment 3: supergene’s effect on male fecundity and mating success

#### (i) Male fecundity

**Figure 5.**
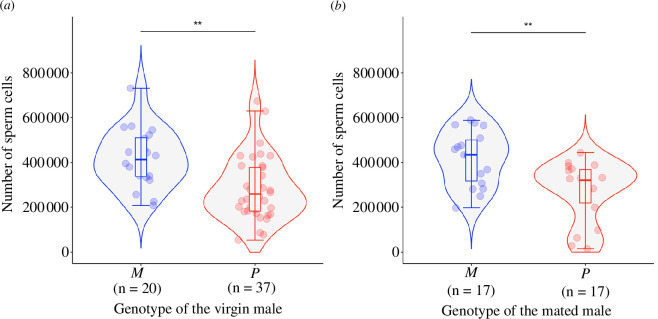
*M* males had higher fecundity than *P* males. Number of sperm cells produced by males (*a*) and transmitted to queens (*b*), according to the genotype of the male. Each dot represents the absolute number of sperm cells collected from a male (or stored by a queen after mating to a male). The horizontal line inside each box shows the median value of sperm cells, and the data inside each box includes the middle 50% of the data. Significance levels: **p < = 0.01.

*M* males had higher fecundity than *P* males. The *M* males produced 1.59 times more sperm cells than the *P* males (median number of sperm cells of virgin *M* males = 412 542; range: 208 328–730 911; median number of sperm cells of virgin *P* males = 259 200; range: 53 656–673 344; electronic supplementary material, table S3*a*, model 13, figure 5*a*). The size of the male did not predict the number of sperm cells he produced (electronic supplementary material, table S3*a*). *P* males also transferred significantly fewer sperm cells than *M* males to queens (median number of sperm cells transferred by *M* males = 434 216; range: 197 597–588 198; median number of sperm cells of virgin *P* males = 321 072; range: 14 859–443 557; electronic supplementary material, table S3*a*, model 13, figure 5*b*), independently of whether this was on a first or second mating event (interaction between the male genotype and his mating status: χ^2^ = 0.17, *p* = 0.68; model 14, figure 5*b*). Queens mated to *M* males stored 1.35 times more sperm cells compared with queens mated to *P* males.

#### (ii) Queens’ mate choice

*MM* queens spent more time near a male odour extract than near the control (electronic supplementary material, table S3*b*, model 15). However, they showed no preference for the odour of *M* males, as they spent similar amounts of time near the cuticular hydrocarbons (CHCs) extracts of *M* or *P* males (electronic supplementary material, table S3*b*, model 16), independently of the male mating status (interaction between male mating status and male genotype: χ^2^ = 0.13, *p* = 0.71; electronic supplementary material, table S3*b*, model 16). Queens spent a median of 21.5 s near the odour of a *P* male (range: 1–176 s) and 25.5 s near the odour of a *M* male (range: 3–184 s).

#### (iii) Male mating success

In artificial swarms, *M* and *P* males were equally likely to mate with *MM* queens (electronic supplementary material, table S3*c*, model 17), irrespective of the male mating status (interaction: χ^2^ = 0.06, *p* = 0.80). Virgin and previously mated males were equally likely to mate (electronic supplementary material, table S3*c*, model 17). Overall, 35% of *M* males and 30% of *P* males successfully mated.

### (d) Experiment 4: supergene’s effect on queen fecundity and colony survival

**Figure 6.**
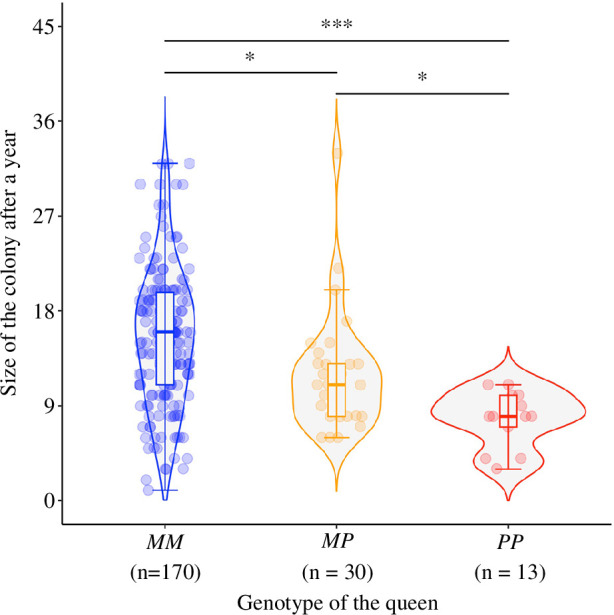
Queens with alternative social supergene genotypes differed in fecundity. Size of 1-year-old colonies (number of workers and pupae), according to the genotype of the founding queens. Each dot represents a 1-year-old colony established by a single queen. The horizontal line inside each box shows the median number of workers, and the data inside each box includes the middle 50% of the data. Significance levels: *p < 0.05, ***p < 0.001 after false discovery rate adjustment for multiple comparisons.

The genotype of the queen influenced whether she was alive and had a brood after a year (electronic supplementary material, table S4, model 18) and, for surviving queens, the size of her colony after a year (electronic supplementary material, table S4, model 18, figure 6). There were significantly more *MM* queens alive with a brood after a year, compared with *PP* queens. Specifically, 42% of the *MM* queens (201 out of 475), 40% of the *MP* queens (35 out of 88) and 27% of the *PP* queens (18 out of 67) were alive after a year. The genotype of the male did not predict the number of workers per colony after a year (electronic supplementary material, table S4, model 19) nor the survival probabilities of the colony (interaction between male and queen genotype: χ^2^ = 1.93, *p* = 0.38, electronic supplementary material, table S4, model 18).

## 4. Discussion

Social, fecundity and dispersal traits coevolve across the tree of life (reviewed in [3]), and covariance between these traits may be genetic or environmental [58]. In *S. invicta*, a social supergene directly affects queen size, dispersal propensity and fecundity [20,33], and influences male fecundity [34]. Here, we show that the independently evolved social supergene of Alpine silver ants [19,23] has similar effects on all of these traits. The convergent properties of two independently evolved social supergenes suggest that a similar genomic evolution led to coordinated changes in social structure, fecundity and dispersal, highlighting the key role of supergenes in linking co-adapted traits and in maintaining intraspecific, discrete complex phenotypes.

Our results show that the derived *Formica* supergene haplotype *P*, found only in polygyne (multi-queen) colonies, is coupled with lower dispersal abilities and reduced fecundity of queens and males, compared with the ancestral supergene haplotype *M*, found mainly in monogyne (single-queen) colonies. At the colony level, previous studies documented that polygyne colonies produce queens and males less frequently and emit fewer queens than monogyne colonies [29,30,32]. At the individual level, we found that *PP* queens and *P* males have phenotypes associated with poorer dispersal abilities and lower fecundity, compared with *MM* queens, *MP* queens and *M* males, respectively. As *P* is dominant for social structure, but recessive for most dispersal traits, and overall less effective for dispersal, *MP* queens and *MM* queens mated to *P* males are likely the primary vehicles for the propagation of the polygyne social form. In both *F. selysi* and *S. invicta*, the derived supergene haplotype causing polygyny is associated with high genetic load, low dispersal ability and low fecundity, and might thus play a causal role in the evolution of polygyny. We discuss each of these findings below.

Individuals carrying the ancestral supergene haplotype, *M*, had better dispersal abilities. Traits influencing the success of queens and males in the early stages of the dispersal process (leaving and moving) include *MM* and *MP* queens having larger and longer wings and more aerodynamic bodies (i.e. smaller ratio of body weight/total wing area) than *PP* queens and *M* males having larger wings than *P* males. Traits influencing the success of queens in the last stage of the dispersal process (settling) are *MM* and *MP* queens being larger and heavier, having higher survival and yielding larger colonies after a year than *PP* queens. Moreover, *M* males produced and transferred more sperm cells than *P* males. Interestingly, we did not find trade-offs among dispersal-related traits and fecundity. Specifically, *MM* queens were the largest, heaviest, most aerodynamic and most fecund of all queens. Similarly, *M* males had larger wings and were more fecund than *P* males. Each year, a higher proportion of monogyne colonies emit alates [29,32], and overall the monogyne social form produces more females [29], females and males that are better dispersers, and females that are more successful at independent colony founding. Such colony- and individual-level effects associated with the ancestral supergene haplotype should lead to higher effective gene flow in the monogyne than in the polygyne social form. In line with this prediction, genome-wide genetic data revealed weaker population genetic structuring in the monogyne social form [42].

The morphological and fertility differences we uncovered between *MP* and *PP* queens is clear empirical proof that the social supergene has direct genetic effects on dispersal-related traits. This is because *PP* and *MP* queens are produced by the same mothers and raised by the same workers within the same polygyne colonies [22], ruling out indirect genetic effects, such as social or environmental effects due to the genotype of other group members [43,44]. Because *MM* queens or *M* males, and *PP* queens or *P* males, are produced by monogyne and polygyne colonies, respectively [19,22,29,30], differences between them might be caused by direct and/or indirect effects of the social supergene. Cross-fostering experiments could help disentangle direct genetic or maternal effects from indirect effects (e.g. [59]).

Some effects of the social supergene were smaller for males than for queens. For instance, the wings of *P* males were 6% smaller than the wings of *M* males, while the wings of *PP* queens were 23% smaller than those of *MM* queens. These differences can be partly explained by selection differing between the sexes. For example, traits related to limited dispersal (smaller body and wing sizes) may have been favoured in polygyne queens, who can be re-adopted within their natal nest [22], but not in males, which remain under strong selection for securing mates in swarms [29].

Lower fecundity of *P* males compared with *M* males and lower survival and fecundity of *PP* queens compared with *MP* or *MM* queens are probably caused by deleterious mutations that tend to accumulate in non-recombining genomic regions [26,60,61]. Recessive deleterious or lethal mutations have been reported in supergenes associated with diverse traits, such as social structure in ants [26,62], life-history traits in seaweed flies [63], mating morphs in ruffs [64] and white-throated sparrows [65], heterostyly in primroses [66] and taillessness in mice [67]. Deleterious mutations result from gene disruptions at inversion breakpoints, genetic drift due to small effective population size during early evolution of the inversion [68] and further accumulation of deleterious mutations by Muller’s ratchet [68,69] (although in rare cases, double crossovers and gene conversion can decrease these negative effects [70,71]). For haplodiploids, recessive deleterious mutations affecting females can persist in heterozygous queens, where they are masked from purifying selection [36,68]. Overall, overdominance and homozygous lethality appear to play a key role in the maintenance of many supergenes [11,72,73].

Non-recombining genomic regions that accumulate deleterious mutations can have surprising detrimental effects cascading across levels of biological organization [16]. In Alpine silver ants, deleterious mutations could have led to reduced body and wing size, and lower fecundity of individuals carrying the inverted non-recombining supergene haplotype. In turn, these detrimental effects may prevent individuals from dispersing and force them to associate in polygyne colonies. Similarly, the inverted supergene haplotype of fire ants is also associated with reduced dispersal [33]. Together, these results raise the possibility that supergene degeneration played a direct causal role in the evolution of queen philopatry and polygyny [16]. Whether supergene degeneration had causal impact on social or dispersal polymorphisms in other systems deserves further investigation.

The *Formica* social supergene has inverted dominance patterns for social organization and dispersal, which likely enables the spread of the polygyne social form. Specifically, the derived haplotype *P* is dominant for social structure (*MP* queens and workers always establish polygyne colonies [22]) but recessive for several dispersal-related traits (*PP* queens have smaller bodies, reduced fecundity and short-range dispersal phenotypes, in comparison to *MP* queens). This suggests that *MP* queens and *MM* queens mated to *P* males likely propagate the polygyne social form. Although in the wild over 70% of eggs laid in polygyne colonies have the *PP* genotype, *PP* workers suffer from very high mortality, as do *PP* queens in the laboratory, so that most surviving queens and workers in polygyne colonies have the *MP* genotype [26]. In addition, about 20% of *MM* queens mate with *P* males in swarms [29]. This cross-produces viable offspring [39,59,74] and occasionally succeeds in establishing incipient colonies that are likely to become polygyne [46].

Transmission ratio distortion by the *P* haplotype tends to destabilize the social polymorphism, and models suggest that strong but incomplete assortative mating by social form is important for its long-term evolutionary persistence [36]. However, the mechanisms accounting for this mating pattern remain unknown. Specifically, *P* and *M* males are both frequent in wild mating swarms [29] and have not been observed to be segregated in space or time [29]. In addition, *MM* queens do not display a strong preference for *M* males [22] or *M* male odour (this study) in laboratory trials. As *M* males have almost twice as many sperm cells as *P* males, it is possible that *M* males mate multiple times, while *P* males do not. It is also possible that *P* males have some competitive advantage when mating within nests. These results show that supergenes have interesting effects on male ants, calling for further studies on their ecology and behaviour.

The *Solenopsis* and *Formica* supergenes have striking similarities in their genomic structure and in the phenotypes they induce. Both systems have extended non-recombining regions (~14 Mbp) including three large inversions and over 600 genes, with the derived haplotype dominantly inducing multi-queen colonies [16,75]. In *S. invicta*, queens established in polygyne colonies are heterozygous and have reduced dispersal abilities [20,33,76]. Based on population genetic data, a model for the dispersal of alternate social forms of *S. invicta* also suggests that the occasional mating of monogyne queens with polygyne males results in polygyne colonies [77]. Strikingly, in both *F. selysi* and *S. invicta*, the derived supergene haplotype, which causes polygyny, is associated with high genetic load, small queen body size, reduced fecundity of queens and males and limited queen dispersal abilities, and seems to rely on the ancestral ‘monogyne’ haplotypes for long-range dispersal.

The convergent properties of the two social supergenes, which evolved independently in the *Formica* and *Solenopsis* lineages [19,23], suggest that a similar genomic evolution led to coordinated changes in both social structure, life-history traits and dispersal strategies, as predicted by theory [14]. The two supergenes are very large, but they show no synteny and differ in gene content, suggesting that similar selection pressures favoured linkage of largely different groups of genes causing similar phenotypic effects [19,23,24,78]. In both lineages, the unusual properties of the underlying genetic systems, including deleterious recessive effects and selfish drive by the derived haplotypes, likely play a direct role in the evolution and maintenance of alternative social forms [16]. More broadly, similarities between two independently evolved supergenes, which control social structure while also influencing dispersal and fecundity, highlight the key role of supergenes in linking behavioural, morphological and physiological traits associated with intraspecific social polymorphisms.

## Data Availability

All data and scripts supporting this study are deposited in Dryad [79 and 80]. Electronic supplementary material is available online [81].
